# Asthma and Respiratory Infections From Birth to Young Adulthood: The Espoo Cohort Study

**DOI:** 10.1093/aje/kwac210

**Published:** 2022-12-14

**Authors:** Abate Bekele Belachew, Aino K Rantala, Maritta S Jaakkola, Timo T Hugg, Jouni J K Jaakkola

**Keywords:** absolute effects, asthma, cohort studies, longitudinal studies, population-based studies, respiratory tract infections

## Abstract

We applied data from a population-based prospective study, the Espoo Cohort Study (*n* = 2,568), to identify the potential susceptibility of persons with asthma to respiratory tract infections (RTIs). Information on the occurrence of asthma and both upper respiratory tract infections (URTIs) and lower respiratory tract infections (LRTIs) was collected with a questionnaire at baseline and at the 6-year and 20-year follow-up studies, and from the Finnish national health registries. We estimated age- and sex-specific incidence rate differences (IRDs) and incidence rate ratios (IRRs) by applying negative binomial regression. Meta-regression was used to summarize the age-specific IRRs from childhood to 27 years of age. Individuals with asthma at any age during the follow-up period had increased risks of both URTIs (adjusted IRD = 72.6 (95% confidence interval (CI): 50.6, 94.7) per 100 person-years; adjusted IRR = 1.27 (95% CI: 1.20, 1.35)) and LRTIs (adjusted IRD = 25.5 (95% CI: 17.9, 33.1); adjusted IRR = 2.87 (95% CI: 2.33, 3.53)) from childhood to young adulthood. In young adulthood, the association between asthma and URTIs was stronger in women than in men, while such an association was not detected for LRTIs. This analysis provides strong evidence that persons with asthma experience more RTIs from preschool age to young adulthood than do those without asthma. Thus, they constitute a susceptible population for RTIs. Women with asthma are at especially high risk.

## Abbreviations

CI: confidence interval
COVID-19: coronavirus disease 2019
EIRR: excess incidence rate ratio
IR: incidence rate
IRD: incidence rate difference
IRR: incidence rate ratio
LRTI: lower respiratory tract infection
REIRI: relative excess incidence rate ratio due to interaction
RTI: respiratory tract infection
URTI: upper respiratory tract infection

Asthma is the most common chronic disease in children and young adults (1). Respiratory tract infections (RTIs) are also among the leading global public health problems (1). Asthma has been reported to increase susceptibility to RTIs, although the mechanisms are poorly understood (2, 3). In previous studies, investigators have reported higher risks of both first-time RTIs and recurrent RTIs in persons with asthma than in healthy subjects among several population groups, including children (4–13), adults (14–18), hospitalized individuals (17, 19, 20), pregnant women (21), and smokers (14). Our previous population-based study showed that atopic diseases, including asthma, rhinitis, and dermatitis, are related to increased risk of RTIs in working-age adults (22).

The results of the previous studies on the association between asthma and RTIs have been inconsistent (6, 15, 16), which may be explained, at least partly, either by their study design or their relatively small sample size. Cross-sectional study designs may restrict causal inference, while results from hospital-based studies may differ from those from population-based studies due to potential selection of cases into hospitals. Based on our systematic search, 8 previous cohort studies had examined potential relationship between bronchial asthma and occurrence of RTIs, as presented in Table 1 (see also Web Figure 1, available at https://doi.org/10.1093/aje/kwac210). Our search strategy is presented in the Web Appendix. These studies covered different age periods, but none of them included as long follow-up of the same population as the present study, the Espoo Cohort Study. None of the previous studies had presented estimates of absolute effects of asthma on the occurrence of respiratory infections in terms of incidence rate differences (IRDs). IRDs are more informative measures of public health impact than incidence rate ratios (IRRs) (23).

**Table 1:** Results From Previous Cohort Studies of the Effect of Asthma on the Risk of Respiratory Tract Infection, by Age Range of the Study Population

**First Author, Year(Reference No.)**	**Study Population**	**Age Range, years**	**Study Focus and Duration of Study Period**	**Definition of Asthma**	**Outcome(s)**	**Effect Estimate**	**95% CI**	**Comment**
Stensballe, 2009 (12)	18,614 twin infants	≤5	Infants born in 1994–2004; 11 years	Asthma (ICD-10)	LRTI: RSV (ICD-10)	HR = 3.00	2.00, 4.70	
Pelton, 2014 (8)	Age ≤4 years: 190,281 persons with asthma and 5,494,451 persons without asthma Age 5–17 years: 1,062,121 persons with asthma and 18,911,630 persons without asthma	≤4 and 5–17	Health claims data from 2007–2010; 4 years	Asthma (ICD-9)	LRTI: pneumonia (ICD-9)	Age ≤4 years: IRR = 3.00 Age 5–17 years: IRR = 3.50	3.00, 3.00 3.40, 3.50	
Frey, 2009 (13)	327 children	≤18	2002–2006; 5 years	Asthma (medical record review, predefined criteria)	URTI: laboratory- confirmed * Streptococcus pyogenes* infection	RR = 1.40	1.12, 1.74	Incidence rates were similar between boys and girls.
Murphy, 2013 (21)	168 pregnant women with asthma and 117 without asthma	18–43	From 12–20 weeks of pregnancy to early postpartum; 6 months	Physician- diagnosed asthma at baseline (self-report)	URTI: self-reported common cold	IRR = 1.77	1.30, 2.42	
Helby, 2017 (14)	105,519 adults	20–109	1991–2014; 23 years	Self-reported asthma at baseline	LRTI: pneumonia (ICD-8 and ICD-10)	HR = 2.03	1.85, 2.22	HRs were similar among ever smokers and never smokers.
Corne, 2002 (15)	76 cohabiting couples	26–50	September–December 1993; 4 months	Atopic asthma at baseline from IUATLD questionnaire	URTI and LRTI: laboratory- confirmed rhinovirus infections, daily symptom diary	OR = 1.15	0.71, 1.87	No difference in the incidence of infection by sex
Ekbom, 2019 (17)	7,284 adults	28–54	2000–2010; 10 years	Asthma at baseline, defined as history of asthma attack or use of medication	LRTI: pneumonia (ICD-10)	HR = 3.35	1.97, 5.70	Women with asthma: IRR = 5.66 (95% CI: 1.26, 25.30)
Liu, 2012 (18)	263,094 adults	≥45	2006–2008; 3 years	Self-reported history of asthma at baseline	Laboratory- confirmed pertussis (ICD-10)	HR = 1.64	1.06, 2.55	

Abbreviations: CI, confidence interval; HR, hazard ratio; ICD-8, *International Classification of Diseases, Eighth Revision*; ICD-9, *International Classification of Diseases, Ninth Revision*; ICD-10, *International Classification of Diseases, Tenth Revision*; IRR, incidence rate ratio; IUATLD, International Union Against Tuberculosis and Lung Disease; LRTI, lower respiratory tract infection; OR, odds ratio; RR, risk ratio; RSV, respiratory syncytial virus; URTI, upper respiratory tract infection.

To address potential susceptibility of subjects with asthma to RTIs, we estimated and compared the occurrence of RTIs between subjects with and without asthma from birth to the age of 27 years. We quantified the differences in the occurrence of RTIs using both relative and absolute measures of effect, estimating both IRRs and IRDs for the relationship of interest.

## METHODS

### Study design and population

This study was a population-based cohort study, the Espoo Cohort Study. The source population included all children living in the city of Espoo, Finland, who were born between January 1, 1984, and March 31, 1989. A parent-administered baseline questionnaire was distributed in March 1991 to a random sample of children in Espoo drawn from the roster of Statistics Finland. A total of 2,568 children whose parents had filled in the questionnaire formed the baseline study population. A 6-year follow-up study of the cohort was conducted in March 1997, with a follow-up rate of 77.3%. In 2010–2011, a 20-year follow-up assessment was conducted, and 1,623 responses were obtained, with a follow-up rate of 63.2%. The age ranges at the baseline, second, and third data collection points were 1–6, 7–13, and 21–27 years, respectively. Details on the study population and follow-up assessments have been published elsewhere (24, 25). The study protocol was approved by the Ethics Committee of Oulu University Hospital, Oulu, Finland.

### Asthma as the determinant of interest

We considered the occurrence of asthma from birth to the end of follow-up as the main determinant of interest. We asked about the presence of physician-diagnosed asthma at baseline and at the 6- and 20-year follow-up assessments. We further confirmed the asthma diagnosis at baseline by contacting the parents, and they all were found to be well-informed about the asthma of their child. In the presence of asthma, we asked about the age of its onset. This information was used to define the first experience of asthma for each member of the cohort and to calculate the individual disease-free person-time. This disease-free person-time was defined from birth to the estimated age of asthma onset or to the end of follow-up for those who did not develop asthma.

### RTIs as the health outcomes of interest

RTIs constituted the outcomes of the present study. Information on RTIs was based on the 3 follow-up questionnaires as well as on register data. In the questionnaires, the occurrence of RTIs during the 12 months preceding each data collection was assessed with the question, “How often did the child/you experience the following infections during the past 12 months?”. The list of options included upper respiratory tract infections (URTIs), including common cold, tonsillitis, sinusitis, and otitis media, and lower respiratory tract infections (LRTIs), including acute bronchitis and pneumonia. The ages of the study subjects during the 3 assessments (baseline, 6 years, and 20 years) corresponded roughly to preschool age, primary school age, and young adulthood. Assessment based on questionnaire data may introduced some recall bias. We were able to also use register-based data that comprised RTIs reported on the basis of objective diagnostic tests.

Information on all RTIs leading to hospitalization between 1984 and 1997 was extracted for each cohort member from the Finnish National Hospital Discharge Register, and information on all RTIs leading to hospitalization or an outpatient visit between 1998 and 2010 was extracted from the Care Register for Health Care. Register-based data were linked to the cohort data using the personal identification number. We extracted register data for all (*n* = 2,568) members of the Espoo Cohort Study who had received the diagnosis up to December 31, 2010. The National Hospital Discharge Register included the dates and causes of all hospital admissions requiring an overnight stay that have occurred since January 1969. The Care Register for Health Care is a continuation of the National Hospital Discharge Register that includes data on hospitalization and health-care visits since 1994. The *International Classification of Diseases* was used to code the diagnoses. We used the Eighth Revision of the *International Classification of Diseases* until 1986, the Ninth Revision between 1987 and 1995, and the Tenth Revision between 1996 and 2010. The disease codes are presented in Web Table 1. We estimated incidence rates (IRs) for the specific RTIs using the number of infections during the follow-up period as the numerator and the time at risk as the denominator. The entire time period was calculated by subtracting the date of birth from the last date of follow-up (i.e., December 31, 2010). We estimated the average episode rate of all RTIs as well as of the specific RTIs for the total study population and for subjects with and without asthma during the study period, as well as during specified time periods using both questionnaire and register-based data.

### Covariates

We included the following covariates as potential confounders, that is, determinants of RTIs: sex (14); atopy (22, 26), which was defined in this study as a history of physician-diagnosed allergic rhinitis and/or eczema; maternal smoking during pregnancy (26, 27); family socioeconomic status at baseline (7, 28, 29); parental atopy/asthma (30); secondhand tobacco smoke exposure (27); indoor mold exposure, defined as the presence of visible mold or a moldy odor (24); duration of pregnancy (26); and duration of breastfeeding (26, 27). Information on these covariates was based on parental report during the baseline assessment. Family socioeconomic status was determined on the basis of the highest level of parental education and occupation at baseline.

### Statistical methods

We assessed the association between asthma (the determinant of interest) and RTIs (the outcome) by comparing the IRs among subjects with and without asthma from childhood to the age of 27 years. We estimated crude and adjusted IRRs and IRDs as measures of effect, applying negative binomial regression with a logit link given overdispersed counts of RTI data. Separate analyses were conducted for RTIs based on the questionnaire and the register data.

We assessed the potential association between asthma at any age and the incidence of RTIs from childhood to young adulthood (to study overall susceptibility), as well as the association when asthma onset preceded RTIs by considering the time period between asthma onset and RTIs (to study causal relationships). For example, we estimated the association between asthma at preschool age and RTIs at the 6-year and/or 20-year follow-ups. Period-specific estimates were pooled using 2-stage fixed-effects meta-analysis assuming fixed effects of asthma across the study period. The *I*^2^ test also showed absence of substantial effect size differences across age groups; hence, the fixed-effect model was reasonable.

The potential effect of the interaction between sex and asthma on the risk of RTIs was studied on an additive scale by estimating the independent and joint effects of sex and asthma on the risk of RTIs. The results are presented as the excess incidence rate ratio (EIRR) for the independent and joint effects for the categories of asthma (A) and sex (B). The relative excess incidence rate ratio due to interaction (REIRI) indicating the departure from additivity of relative risks was quantified as REIRI = EIRR (AB) − EIRR (A) − EIRR (B).

The 95% confidence interval (CI) for REIRI was estimated using the method of variance estimates recovery (31), and the null value corresponds to a statistical significance level of *P* = 0.05. All analyses were conducted using Stata, version 16 (StataCorp LLC, College Station, Texas).

## RESULTS

### Characteristics of the study population

Characteristics of the study population are presented in Table 2. The number of subjects was 2,568 at baseline and subsequently 1,984 in the 6-year follow-up study and 1,623 in the 20-year follow-up study, giving follow-up rates of 77.3% and 63.2%, respectively. The baseline characteristics of study subjects with and without asthma are described in Web Table 2.

**Table 2:** Characteristics of the Study Population at Baseline and in 6-Year and 20-Year Follow-up Surveys, Espoo Cohort Study, 1991–2011^a^

	**Assessment**					
	**Baseline**		**6-Year**		**20-Year**	
**Baseline Characteristic**	**No.**	**%**	**No.**	**%**	**No.**	**%**
No. of subjects	2,568	100	1,984	77.3	1,623	63.2
Age, years^b^	3.6 (1.8)		10.1 (1.8)		23.0 (1.8)	
Sex						
Female	1,257	48.9	982	49.5	869	53.5
Male	1,311	51.1	1,002	50.5	754	46.5
Duration of breastfeeding, months						
<4	481	19.3	346	18.0	289	18.3
4–7	670	26.9	511	26.5	420	26.6
≥8	1,337	53.7	1,071	55.5	869	55.1
Duration of pregnancy, weeks						
<37	195	7.8	148	7.7	99	6.2
≥37	2,301	92.2	1,782	92.3	1,488	93.8
Maternal smoking						
No	2,199	85.8	1,722	86.9	1,415	87.4
Yes	364	14.2	260	13.1	204	12.6
Family socioeconomic status						
Low	667	26.1	498	25.2	371	22.9
Medium/high	1,889	73.9	1,478	74.8	1,246	77.1
Atopy						
No	2,276	88.6	1,760	88.7	1,444	89.0
Yes	292	11.4	224	11.3	179	11.0
Parental atopy						
No	1,697	66.1	1,327	66.9	1,083	66.7
Yes	871	33.9	657	33.1	540	33.3
Secondhand cigarette smoke exposure						
No	2,171	84.5	1,690	85.2	1,388	85.5
Yes	397	15.5	294	14.8	235	14.5
Indoor mold exposure						
No	2,428	94.6	1,867	94.1	1,540	94.9
Yes	138	5.4	117	5.9	83	5.1

^a^ There were missing values for duration of breastfeeding (*n* = 79), duration of pregnancy (*n* = 72), maternal smoking (*n* = 5), family socioeconomic status (*n* = 12), and indoor mold exposure (*n* = 2); percentages were calculated using the respective number of subjects at each follow-up point as the denominator.

^b^ Values are expressed as mean (standard deviation).

### Incidence of RTIs

**Figure 1:**
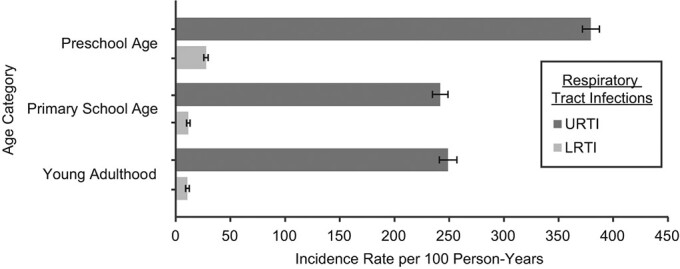
Average incidence rate of upper respiratory tract infection (URTI) and lower respiratory tract infection (LRTI) per 100 person-years in 3 age periods (preschool (ages 1–6 years), primary school (ages 7–13 years), and young adulthood (ages 21–27 years)), Espoo Cohort Study, 1991–2011. Bars, 95% confidence intervals.

The IR of RTIs was higher at preschool age than at primary school age and in young adulthood (Figure 1). Hospitalizations due to RTIs also showed higher numbers of episodes of RTIs during preschool age as compared with subsequently older age groups (Table 3). Sex-specific IRs of RTIs are presented in Web Tables 3 and 4. The effect of sex was found to be different by age of the study subjects. At preschool age, the incidence of URTIs was higher in boys than in girls, whereas at subsequent ages (primary school age and young adulthood), the incidence was higher in girls than in boys. However, the incidence of LRTIs was higher in boys than in girls from childhood through young adulthood.

**Table 3:** Numbers of Cases and Incidence Rates of Upper and Lower Respiratory Tract Infection According to Age at Health-Care Visit (*n* = 2,568), Espoo Cohort Study, 1991–2011^a^

	**Age at Health-Care Visit, years**									
	**≤6**		**7–13**		**14–20**		**21–27**		**Total**	
**Type of Infection**	**No. of** **Episodes**	**IR** ^**b**^	**No. of** **Episodes**	**IR^**b**^**	**No. of** **Episodes**	**IR^**b**^**	**No. of** **Episodes**	**IR^**b**^**	**No. of** **Episodes**	**IR^**b**^**
Upper respiratory tract infection	219	142.57	48	31.25	156	101.56	58	37.76	481	78.78
Common cold	12	7.81	0	0	2	1.30	2	1.30	16	2.62
Acute tonsillitis	17	11.06	7	4.56	59	28.41	21	13.67	104	17.03
Acute sinusitis	33	21.48	27	17.57	45	29.30	13	8.46	118	19.32
Acute laryngitis/tracheitis	58	37.76	4	2.60	3	1.95	0	0	65	10.65
Acute otitis media	60	39.06	2	1.30	0	0	0	0	62	10.15
Unspecified	39	25.39	8	5.20	47	30.59	22	14.32	116	19.00
Lower respiratory tract infection	112	72.92	29	18.88	74	48.18	22	14.32	237	38.82
Acute bronchitis	22	14.32	7	4.56	8	5.20	4	2.60	35	5.73
Viral pneumonia	6	3.90	2	1.30	0	0	0	0	8	1.31
Pneumococcal pneumonia	1	0.65	0	0	0	0	0	0	1	0.16
Other bacterial pneumonia	1	0.65	3	1.95	40	26.04	4	4.56	51	8.35
Pneumonia of another specified organism	2	1.30	0	0	0	0	0	0	2	0.32
Unspecified pneumonia	80	52.08	14	9.12	26	16.93	11	7.16	131	21.46

Abbreviation: IR, incidence rate.

^a^ Information on infections was obtained from the National Hospital Discharge Registry (1991–1997) and the Care Register for Health Care (1998–2011).

^b^ Number of episodes per 10,000 person-years.

### Asthma and RTI incidence from childhood to young adulthood

We present the IRs of RTIs by asthma status in Web Tables 5–11. The IR of RTIs was found to be higher in asthmatics than in nonasthmatics from childhood to young adulthood. Consistent with the results from questionnaire-based information also, hospital-based RTIs were more common in children with asthma than in nonasthmatic children (Web Table 12).

### Association between asthma and RTIs during the entire study period

Subjects with asthma had about 73 (adjusted IRD = 72.6, 95% CI: 50.6, 94. 7) and 26 (adjusted IRD = 25.5, 95% CI: 17.9, 33.1) excess episodes of URTIs and LRTIs per 100 person-years compared with those who had no asthma, respectively (Table 4). Consistently, when we took into account the time period between the onset of asthma and RTIs, asthma at preschool age was associated with higher IRs of both URTIs (adjusted IRD = 73.5, 95% CI: 42.1, 104.9) and LRTIs (adjusted IRD = 19.3, 95% CI: 8.5, 30.1) at school age or in young adulthood (Table 4).

**Table 4:** Adjusted Incidence Rate Differences for Upper and Lower Respiratory Tract Infection Between Subjects With and Without Asthma From Childhood to Young Adulthood, Espoo Cohort Study, 1991–2011

		**URTI**		**LRTI**	
**Age at Asthma Onset and** **Age at Infection, years**	**No. of** **Subjects**	**IRD** ^**a**^	**95% CI**	**IRD** ^**a**^	**95% CI**
Never had asthma	2,253	1.0	Referent	1.0	Referent
Preschool age (≤6 years)^b^	167				
7–13		73.0	33.4, 112.6	22.2	7.9, 36.6
21–27		72.5	22.7, 122.4	15.9	−0.2, 31.9
7–13 or 21–27		73.5	42.1, 104.9	19.3	8.5, 30.1
Primary school age (7–13 years)^b^	93				
21–27		51.0	−6.2, 108.7	15.2	−4.4, 34.8
Ever having asthma (≤27 years)	315				
1–6		92.9	50.2, 135.5	38.8	23.4, 54.3
7–13		68.7	39.6, 97.7	16.9	7.5, 26.3
21–27		67.4	32.4, 102.4	20.1	7.3, 32.9
1–6, 7–13, or 21–27		72.6	50.6, 94.7	25.5	17.9, 33.1

Abbreviations: CI, confidence interval; IRD, incidence rate difference; LRTI, lower respiratory tract infection; URTI, upper respiratory tract infection.

^a^ Difference in incidence rates per 100 person-years. The effect estimates were adjusted for sex, personal atopy, maternal smoking, family socioeconomic status, parental atopy/asthma, secondhand smoke exposure, indoor mold exposure at baseline, duration of pregnancy, and duration of breastfeeding.

^b^ Onset of asthma before the occurrence of infection was applied in the analyses. Fifty-five subjects developed asthma at age 14–27 years.

**Figure 2:**
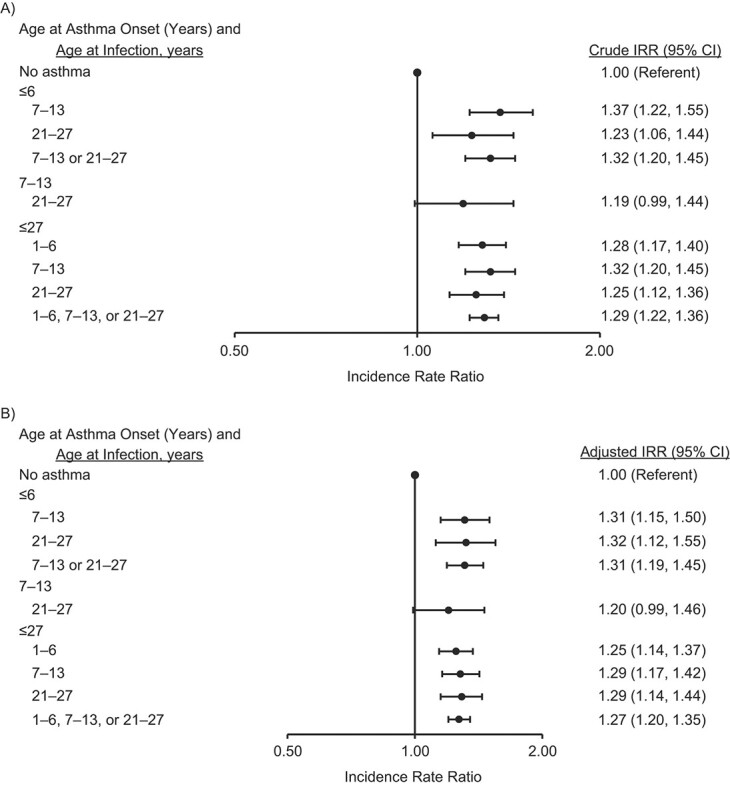
Crude (A) and adjusted (B) incidence rate ratios (IRRs) for the association between asthma and incident upper respiratory tract infection from childhood to young adulthood, Espoo Cohort Study, 1991–2011 (167, 93, and 315 subjects had asthma at ages ≤6 years, 7–13 years, and ≤27 years, respectively). The effect estimates were adjusted for sex, personal atopy, maternal smoking, family socioeconomic status, parental atopy/asthma, second-hand smoke exposure, indoor mold exposure at baseline, duration of pregnancy, and duration of breastfeeding. Bars, 95% confidence intervals (CIs).

**Figure 3:**
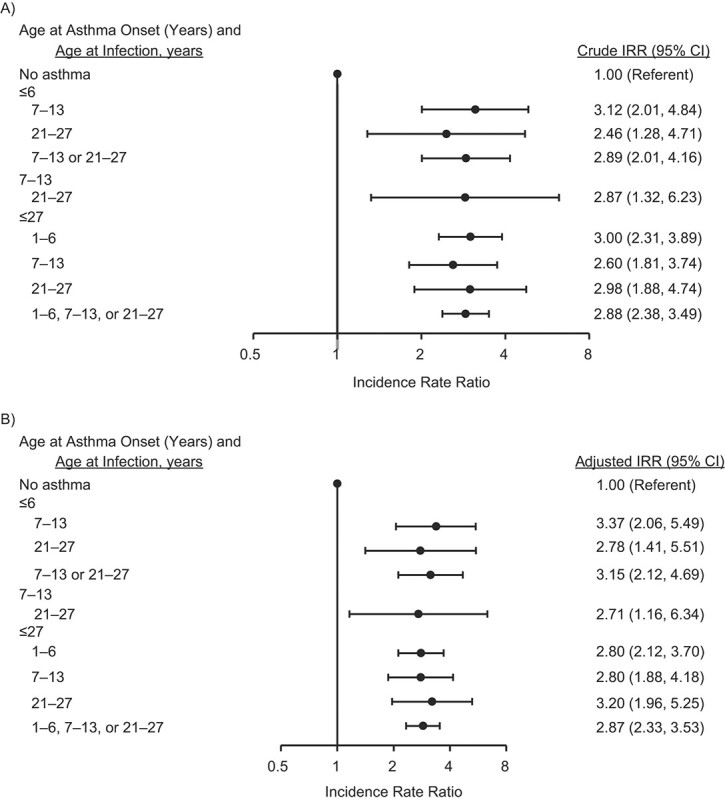
Crude (A) and adjusted (B) incidence rate ratios (IRRs) for the association between asthma and incident lower respiratory tract infection from childhood to young adulthood, Espoo Cohort Study, 1991–2011 (167, 93, and 315 subjects had asthma at ages ≤6 years, 7–13 years, and ≤27 years, respectively). The effect estimates were adjusted for sex, personal atopy, maternal smoking, family socioeconomic status, parental atopy/asthma, second-hand smoke exposure, indoor mold exposure at baseline, duration of pregnancy, and duration of breastfeeding. Bars, 95% confidence intervals (CIs).

The adjusted effect estimates for the relationship between asthma and URTIs and LRTIs are presented in Figures 2 and 3, respectively. Asthma at any age was associated with a 1.3 times’ increased IR of URTIs (adjusted IRR = 1.27, 95% CI: 1.20, 1.35) and a 2.9 times’ increased rate of LRTIs (adjusted IRR = 2.87, 95% CI: 2.33, 3.53). This association was consistent also when taking into account the time period between the onset of asthma and RTIs, which showed that having asthma at preschool age increased the risk of URTIs (adjusted IRR = 1.31, 95% CI: 1.19, 1.45) and LRTIs (adjusted IRR = 3.15, 95% CI: 2.12, 4.69) at school age or in young adulthood (Figures 2 and 3).

We present the adjusted estimates for the effect of asthma on the risk of hospital visits due to RTIs in Web Tables 13 and 14.

### Joint effect of sex and asthma on the risk of RTIs

Results for the joint effect of sex and asthma on the risk of URTIs from childhood to young adulthood are presented in Table 5. The joint effect of asthma and female sex on the risk of URTIs showed an adjusted IRR of 1.73 (95% CI: 1.49, 2.02), which was considerably higher than their independent effects on an additive scale (REIRI = 0.35, 95% CI: 0.04, 0.66; *P* = 0.025). In other words, when compared with the risk that would have been expected by summing independent effects of asthma and female sex, there was a 35% (95% CI: 4, 66) excess risk associated with their joint effect. However, this was not observed in preschool-age or primary-school–age groups (data not shown). Furthermore, the effect of asthma on the risk of LRTIs appeared not to be different between boys and girls during childhood or young adulthood (data not shown).

**Table 5:** Independent and Joint Effects of Sex and Asthma on the Incidence Rate of Upper Respiratory Tract Infection During Young Adulthood (*n* = 1,623), Espoo Cohort Study, 1991–2011

			**Effect Estimate**									
**Asthma Status**	**Sex**	**No. of Subjects**	**IR** ^**a**^	**95% CI**	**Crude IRR**	**95% CI**	**Adjusted IRR** ^**b**^	**95% CI**	**EIRR** ^**b**^	**95% CI**	**REIRI** ^**b**^	**95% CI**
No	Male	640	2.18	2.06, 2.29	1.00	Referent	1.00	Referent	1.00	Referent		
Yes	Male	114	2.34	2.07, 2.64	1.08	0.91, 1.27	1.13	0.95, 1.35	0.13	0.13, 0.35		
No	Female	744	2.68	2.56, 2.80	1.23	1.13, 1.35	1.25	1.14, 1.37	0.25	0.14, 0.37		
Yes	Female	125	3.71	3.38, 4.07	1.71	1.48, 1.97	1.73	1.49, 2.02	0.73	0.49, 1.02	0.35	0.04, 0.66

Abbreviations: CI, confidence interval; EIRR, excess incidence rate ratio; IR, incidence rate; IRR, incidence rate ratio; REIRI, relative excess incidence rate due to interaction.

^a^ Number of episodes per person-year.

^b^ Adjusted for sex, personal atopy, maternal smoking during pregnancy, family socioeconomic status, parental atopy/asthma, secondhand smoke exposure, indoor mold exposure at baseline, and durations of pregnancy and breastfeeding.

## DISCUSSION

### Main findings

Our findings, obtained using data from a longitudinal study and including information from both questionnaires and registers, provide new evidence that asthma increases the risk of RTIs throughout the period from childhood to the teenage years and young adulthood. Asthma was associated with up to a 1.3-fold increased risk of URTIs and a 2.9-fold increased risk of LRTIs. Subjects with asthma experienced, on average, 0.7 excess episodes of URTIs and 0.2 excess episodes of LRTIs per year compared with subjects without asthma. In particular, we observed that individuals with asthma had an increased risk of RTIs when asthma onset preceded RTIs. We also found that asthma and female sex had a synergistic effect on the risk of URTIs during young adulthood.

### Synthesis with previous knowledge

Previously evidence has been reported showing an effect of RTIs on the onset of asthma. In the previous analysis of the Espoo Cohort Study, we showed that both URTIs (adjusted hazard ratio = 1.64, 95% confidence interval (CI): 1.22, 2.19) and LRTIs (adjusted hazard ratio = 2.11, 95% CI: 1.48, 3.00) in early childhood were strong predictors of asthma incidence up to young adulthood (ages 20–27 years) (25). However, a new hypothesis suggesting that asthma increases susceptibility to RTIs has also been recently raised. In a 2014 review, Juhn (2) reported 2 types of relationships between RTIs and asthma. Our previous study (25) and the current findings complement each other in addressing the hypothesis that bronchial asthma is related to respiratory infections: 1) having respiratory infections increases the risk of developing bronchial asthma; 2) having asthma predisposes a person to more easily catch respiratory infections compared with healthy subjects. Though the mechanisms are not yet fully understood, both immunological dysfunction and structural airway alterations observed in asthmatic patients are likely to play a role in such susceptibility (2). The review also suggested that the severity of asthma is not likely to influence the effect of asthma on the risk of RTIs.

We conducted a systematic literature search concerning the effect of asthma on the risk of RTIs (see Web Appendix). Of 8 studies, with a median follow-up duration of 7.1 years, 7 found that subjects with asthma have a higher incidence of various RTIs than subjects without asthma, which is consistent with our findings. However, a 2002 cohort study by Corne et al. (15) showed an absence of any meaningful difference in the risk of rhinovirus infection related to asthma and nonasthma groups. This difference in results could be explained by the rather small sample size in that study.

We found the average IR of URTIs to be approximately 4 episodes per person-year at preschool age and more than 2 episodes per person-year at primary school age and in young adulthood. Hospitalizations due to RTIs showed a similar decreasing trend with age, that is, children at preschool age had a higher IR of RTIs than older age groups. Consistently, 2 previous studies found a decreasing frequency of RTIs with increasing age (10, 32). A relatively naive immune system at preschool age could explain, at least partly, this higher rate of infections during preschool age (33).

In the present study, the IR of URTIs was estimated to be higher among boys than among girls at preschool age, while girls were found to have a higher IR than boys at primary school age and in young adulthood. Furthermore, we found that boys had a higher IR of LRTIs than girls from early childhood to young adulthood. Our findings are consistent with previous studies from Denmark (32) and Israel (34). In a systematic review based on studies conducted up to September 2006, Falagas et al. (35) reported a higher frequency of LRTIs in boys than in girls among both children and adults. The frequency of URTIs was greater among girls than among boys (35).

We found that sex modified the relationship between asthma and the incidence of URTIs in young adulthood. However, such modification was not identified in relation to LRTIs. To our knowledge, this is the first study to have reported a modifying effect of sex on the relationship between asthma and RTIs. The stronger effect of asthma on susceptibility to infections in girls compared with boys in young adulthood could be explained by the differences in airway microbiota (36), by sex hormones (33), and/or by different types of immune responses (33).

### Validity of results

This was a 20-year population-based cohort study that was based on a random sample of children at baseline. We achieved rather good follow-up rates—77.3% at the 6-year follow-up and 63.2% at the 20-year follow-up. There were no major differences in the characteristics of the baseline population or the study population at the 2 subsequent follow-ups (Table 2), so any major selection bias was unlikely.

Information on bronchial asthma was based on parent-reported or (in the last phase) self-reported information on physician-diagnosed asthma. Nwaru et al. (37) compared Finnish questionnaire-based information on physician-diagnosed asthma with information from the Finnish asthma medication reimbursement register in 5-year-old children and showed that a parental report about physician-diagnosed asthma is a valid tool for epidemiologic studies. We conducted further verification of asthma during the baseline study by contacting those parents who reported physician-diagnosed asthma, and all parents were found to be well-informed about the presence of asthma in their children. Further, it is realistic to assume that young adults at the 20-year follow-up were well aware of their diagnosed asthma. The Finnish health-care system provides comprehensive health insurance coverage for the entire population and provides special reimbursement for the cost of asthma medications, which is an incentive for people to undergo appropriate diagnostic investigations to have their asthma clinically diagnosed. We further considered the age of onset of asthma when categorizing a person as having asthma or not at each assessment point, which increased the reliability of incidence estimates of asthma in each study period.

The information on the occurrence of RTIs in the past 12 months was based first on parents’ reporting and then on self-reporting. Difficulty in recalling the exact frequency and type of respiratory infections may have caused some misclassification of the outcomes. Some symptoms and signs of asthma could have been interpreted as respiratory infections, especially by the parents or cohort members. This would be a problem, especially among subjects with untreated asthma or poorly controlled asthma. Because of good population-wide access to public health services, availability of subsidized asthma medications to all subjects with bronchial asthma, and awareness of asthma on the part of health-care personnel and the general population, the proportion of subjects with poorly controlled asthma was relatively low in Finland at the time of this data collection. In 1994–2004, Finland pursued a national asthma program that had a strong influence on awareness of asthma, as well as on better prevention and treatment of asthma. The achievements of this program are documented in the scientific literature (37). The national campaign was continued and expanded to both allergies and asthma in 2008–2018 (38).

Our findings were strengthened by our ability to use the information on RTIs from 2 complete and reliable data sources—the National Hospital Discharge Register up to 1997 and the Care Register for Health Care from 1998 to 2011. It is possible that some clinicians were more inclined to diagnose a respiratory infection among subjects with asthma and asthma-related conditions. However, the broad availability of diagnostic tools, such as viral and bacterial diagnostics, general measures of infection diagnostics, and radiographs, was likely to have reduced this type of information bias. Especially severe respiratory infections requiring hospital care were carefully diagnosed, and information bias was unlikely.

The results from analyses that applied data on RTIs from the 2 sources of data—the questionnaire and the hospital register—were largely consistent. Information on hospital admissions that required an overnight stay was available for the whole follow-up period, while information on health-care visits was available only from 1998 onwards. Therefore, the reliability of the information on RTIs may have differed according to the studied age group.

We conducted the following sensitivity analyses. First, we included only RTIs that required an overnight stay in the hospital; then, we included RTIs based on information on both overnight stays and outpatient visits. We found that the relationships between asthma and RTIs were similar with both types of information on infections. Therefore, we report only findings that include the full register-based information on RTIs. Estimates of the effect of the relationship between asthma at primary school age and secondary school age and health-care visits due to RTIs should be interpreted with caution, because there were no cases or just a few cases across the categories of subjects based on their asthma (Web Tables 12–14).

Furthermore, parental reports are likely to indicate symptomatic infections, that is, clinical infections, which are more serious than infections with few or no symptoms. Because symptomatic infections are usually more severe illnesses and place a greater disease burden on the health-care system, we consider these results to be relevant.

We were able to adjust for several potential confounders, including parental and study subjects’ characteristics and environmental exposures at home. Some of our study design features may be unique and related to the Finnish health-care system and exposure environment. Given the almost similar underlying biology of asthma in all populations across different settings (2, 39), our findings are likely to be applicable to other settings.

### Conclusions

In conclusion, our population-based cohort study following the same population from perinatal care to the age of 27 years showed that having asthma increases the risk of catching RTIs. In addition to estimating IRRs as relative measures of effect, we estimated IRDs as absolute measures of effect, providing effect estimates suitable for the evaluation of the public health impact of asthma on respiratory infections. The effect of asthma was found to be stronger in women than in men in young adulthood, particularly for URTIs. Thus, awareness of increased susceptibility to RTIs should be raised among persons with a diagnosis of asthma, as well as among those treating them, in order to improve the prevention and treatment of RTIs. Although the data collection for these analyses was conducted before the coronavirus disease 2019 (COVID-19) pandemic, our results imply that subjects with asthma are more vulnerable to catching respiratory infections. This is most likely also true for COVID-19 infection. Therefore, subjects with asthma may benefit from preventive measures implemented to combat the COVID-19 pandemic.

## Supplementary Material

Web_Material_kwac210
