# Supplementary material for: Asthma and Respiratory Infections From Birth to Young Adulthood: The Espoo Cohort Study
Source: Am J Epidemiol. 2022 Dec 14;192(3):408–19. doi: 10.1093/aje/kwac210 (PMC9985109; doi:10.1093/aje/kwac210)
Supplement: Web_Material_kwac210 [file web_material_kwac210.pdf]

WEB MATERIAL

Asthma and Respiratory Infections From Birth to Young Adulthood: The Espoo Cohort Study

Abate Bekele Belachew, Aino K. Rantala, Maritta S. Jaakkola, Timo T. Hugg, and Jouni J. K. Jaakkola

Contents:

Web Appendix

Web Figure 1

Web Tables 1–14

Web Appendix

Systematic Review of Literature About the Effects of Asthma on the Risk of Respiratory Tract Infections and Literature Search Strategy

A comprehensive search of three databases including PubMed, Web of Science, and Scopus, from their inception to November 04, 2021, English language and with no age restriction, was conducted to elaborate on the relation between the presence of asthma and occurrence of respiratory infections. The search strategy was designed after getting a consultation from an experienced librarian. Controlled vocabulary supplemented with keywords was used to search for the literature on the topic. Three specific keywords (asthma, risk, and respiratory tract infections) are used for literature search using Boolean logic (AND/OR). The specific terms used are described as follows:

1. Asthma and related search terms (asthma, hypersensitivity, atopic dermatitis, and allergic rhinitis)
2. Risk or comorbidity
3. Respiratory tract infection and related search terms (respiratory infection(s), respiratory morbidity, pneumococcal infections, invasive pneumococcal disease)
4. (1 AND 2); AND (3)
5. Limit 4 to English, and journal articles
6. From 4, exclude subject areas except "Medicine", "Immunology and Microbiology", "Biochemistry, Genetics, and Molecular Biology", "Nursing", "Multidisciplinary" and "Health Professions"

This search returned 8,369 potential articles which met the search criteria. The inclusion criteria included: 1) Studies conducted human population; 2) Any epidemiologic study design; 3) Studies reported effect estimates for the association between asthma and RTIs. ABB independently reviewed these articles. The selection process is presented schematically as follows (Web Figure 1).

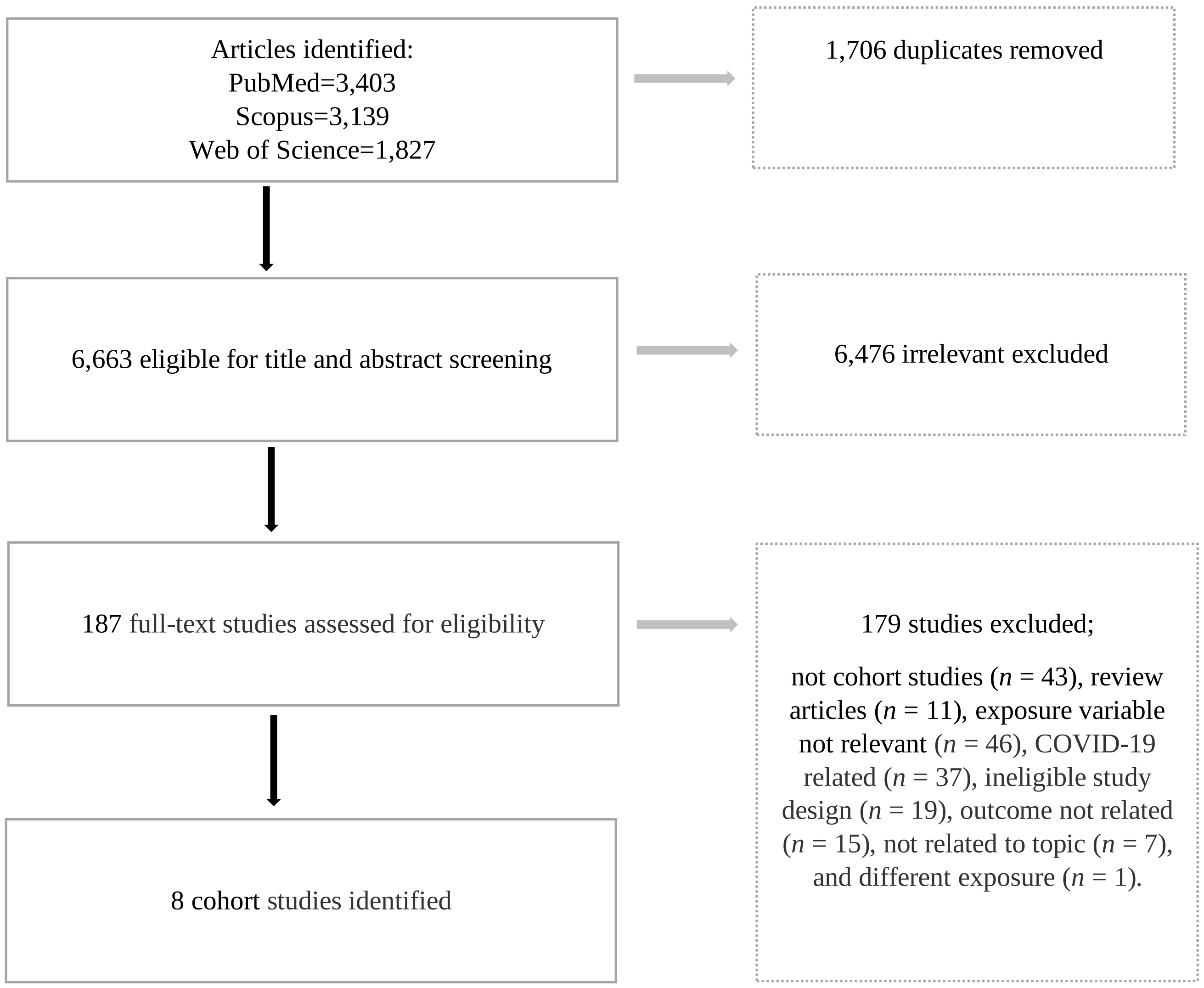

Web Figure 1. Article selection process

Web Table 1. ICD Codes and Diagnoses

Diagnosis	ICD-8	ICD-9	ICD-10
**Upper respiratory tract infections**			
Acute nasopharyngitis		460.0A	J00
(Common cold)			
Acute sinusitis		461.0A, 461.9X	J01.0, J01.1, J01.2, J01.4, J01.8, J01.9
Acute tonsillitis/pharyngitis		463.09 463.0A	J02.0, J02.9, J03.0, J03.9
Acute laryngitis and tracheitis		464.0A, 464.01, 464.09, 464.2A, 464.3A	J04.0, J05.1
Acute upper respiratory infections of unspecified sites		465.99, 465.9X	J06.9, J06.89
**Lower respiratory tract infections**			
Acute bronchitis		466.0A, 466.1A, 466.99	J20.0, J20.9, J21.9
Viral pneumonia		480.1A, 480.8X, 480.9X	J12.9
Pneumococcal pneumonia		481.0A	
Other bacterial pneumonia	482.98		J15.9
Pneumonia due to another specified organism		483.0A, 483.0X	
Unspecified pneumonia	486.09	485.09, 485.9X, 486.11	J18.9

ICD – International Classification of Disease

Web Table 2. Baseline Characteristics of the Study Population for Ever Having Asthma, Espoo Cohort Study, 1991–2011

Variable [a]		Asthma (*n* = 315)	No asthma (*n* = 2,253)	*P* Value [b]
Age, years [c]		3.8 (±1.8)	3.6 (±1.8)	>0.05 (t-test)
Duration of breastfeeding, weeks [c]		8.6 (±6.0)	8.1 (±4.9)	>0.05 (t-test)
Sex				
	Female	150 (47.6)	1,107 (49.1)	0.614
	Male	165 (52.4)	1,146 (50.9)	
Duration of pregnancy, weeks				
	≥37	281 (92.1)	2,020 (92.2)	0.969
	<37	24 (7.9)	171 (7.8)	
Atopy				
	No	226 (71.8)	2,050 (91.0)	<0.001
	Yes	89 (28.2)	203 (9.0)	
Parental atopy				
	No	167 (53.0)	1,530 (67.9)	<0.001
	Yes	148 (47.0)	723 (32.1)	
Family socioeconomic status				
	Low	91 (29.1)	576 (25.7)	0.200
	High/medium	222 (70.9)	1,667 (74.3)	
Indoor mold exposure				
	No	291 (92.1)	2,137 (94.9)	0.060
	Yes	24 (7.9)	114 (5.1)	
Second-hand cigarette smoke exposure				
	No	275 (87.3)	1,896 (84.2)	0.148
Yes		40 (12.7)	356 (15.8)	

[a] There were missing values in duration of breastfeeding (*n* = 79), duration of pregnancy (*n* = 72), maternal smoking (*n* = 5), family socioeconomic status (*n* = 12), and indoor mold exposure (*n* = 2).
[b] Chi-square test unless indicated
[c] Mean (±standard deviation)

Web Table 3. Incidence Rates of Upper Respiratory Tract Infections and Lower Respiratory Tract Infections by Sex from Preschool Age to Young Adulthood, Espoo Cohort Study, 1991–2011[a]

Infection	Sex	Age: 1–6 Years		Age: 7–13 Years		Age: 21–27 Years	
		IR (95% CI)	IRR (95% CI)	IR (95% CI)	IRR (95% CI)	IR (95% CI)	IRR (95% CI)
URTIs	Female	3.65 (3.54, 3.76)	1.00	2.54 (2.44, 2.64)	1.00	2.82 (2.72, 2.94)	1.00
	Male	3.94 (3.84, 4.06)	1.08 (1.03,1.12)	2.37 (2.27, 2.46)	0.93 (0.88, 0.98)	2.20 (2.10, 2.31)	0.78 (0.73, 0.83)
Common cold	Female	2.58 (2.49, 2.67)	1.00	2.03 (1.94, 2.12)	1.00	2.48 (2.37, 2.59)	1.00
	Male	2.74 (2.65, 2.83)	1.05 (1.01, 1.11)	1.84 (1.76, 1.93)	0.91 (0.85, 0.97)	1.88 (1.78, 1.98)	0.78 (0.73, 0.84)
Acute tonsillitis	Female	0.09 (0.07, 0.1)	1.00	0.14 (0.12, 0.17)	1.00	0.09 (0.08, 0.12)	1.00
	Male	0.10 (0.08, 0.12)	1.11 (0.89, 1.5)	0.14 (0.12, 0.17)	0.89 (0.69, 1.14)	0.09 (0.07, 0.12)	0.96 (0.68, 1.33)
Acute sinusitis	Female	0.07 (0.05, 0.08)	1.00	0.16 (0.14, 0.19)	1.00	0.27 (0.23, 0.30)	1.00
	Male	0.06 (0.05, 0.08)	0.95 (0.7, 1.3)	0.17 (0.15, 0.20)	1.05 (0.84, 1.31)	0.19 (0.16, 0.22)	0.70 (0.56, 0.86)
Acute otitis media	Female	0.90 (0.84, 0.95)	1.00	0.21 (0.18, 0.24)	1.00	0.07 (0.05, 0.09)	1.00
	Male	1.03 (0.97, 1.09)	1.14 (1.06, 1.24)	0.23 (0.19, 0.26)	1.1 (0.9, 1.32)	0.05 (0.03, 0.06)	0.64 (0.41, 0.99)
LRTIs	Female	0.23 (0.2, 0.25)	1.00	0.10 (0.08, 0.12)	1.00	0.13 (0.11, 0.16)	1.00
	Male	0.33 (0.3, 0.36)	1.44 (1.24, 1.68)	0.14 (0.11, 0.16)	1.32 (1.01, 1.72)	0.11 (0.09, 0.14)	0.82 (0.61, 1.09)
Acute bronchitis	Female	0.19 (0.17, 0.22)	1.00	0.08 (0.06, 0.10)	1.00	0.12 (0.1, 0.15)	1.00
	Male	0.28 (0.26, 0.31)	1.48 (1.26, 1.75)	0.11 (0.09, 0.14)	1.37 (1.02, 1.86)	0.09 (0.07, 0.12)	0.77 (0.56, 1.06)
Pneumonia	Female	0.04 (0.02, 0.05)	1.00	0.02 (0.01, 0.03)	1.00	0.01 (0.01, 0.02)	1.00
	Male	0.04 (0.03, 0.05)	1.21 (0.8, 1.84)	0.02 (0.01, 0.04)	1.12 (0.60, 2.12)	0.02 (0.01, 0.03)	1.44 (0.63, 3.37)

IR – Incidence rate per person-year; CI – Confidence interval; IRR – Incidence rate ratio. URTIs – Upper Respiratory Tract Infections; LRTIs – Lower Respiratory Tract Infections.

[a] Incidence rate ratios (IRRs) are calculated from IRs among men divided by IRs among women.

Web Table 4. Incidence Rates of Hospitalization and Health Care Visit due to Respiratory Tract Infections by Sex of Study Subjects from Preschool age to Young Adulthood, Espoo Cohort Study, 1991–2011 [a]

Infection	Sex	Age: 0–6 Years		Age: 7–13 Years		Age: 14–20 Years		Age: 21–27 Years	
		**IR (95% CI)**	**IRR (95% CI)**	**IR (95% CI)**	**IRR (95% CI)**	**IR (95% CI)**	**IRR (95% CI)**	**IR (95% CI)**	**IRR (95% CI)**
URTIs	Female	11.27 (9.0, 13.94)	1.00	1.6 (0.82, 2.8)	1.00	7.3 (5.49, 9.49)	1.00	4.64 (3.23, 6.45)	1.00
	Male	17.04 (14.27, 20.17)	1.51 (1.14, 2.0)	4.57 (3.21, 6.34)	2.87 (1.46, 6.07)	12.8 (10.45, 15.6)	1.76 (1.26, 2.49)	2.92 (1.85, 4.39)	0.63 (0.36, 1.09)
LRTIs	Female	6.23 (4.57, 8.28)	1.00	1.99 (1.11, 3.28)	1.00	5.57 (4.01, 7.53)	1.00	2.12 (1.21, 3.45)	1.00
	Male	8.26 (6.38, 10.53)	1.32 (0.89, 1.97)	1.78 (0.97, 2.98)	0.89 (0.4, 1.99)	4.07 (2.78, 5.78)	0.73 (0.45, 1.19)	0.76 (0.28, 1.66)	0.36 (0.12, 0.97)

IR – Incidence rate per 1000 person-year; CI – Confidence interval; IRR – Incidence rate ratios; URTIs – Upper respiratory tract infections; LRTIs – Lower respiratory tract infections.

[a] Given a few numbers of cases, data on specific infections is not presented and this may also have effect on the current estimates, thus conclusions should be made considering this limitation. Incidence rate ratios (IRRs) are calculated from IRs among men divided by IRs among women.

Web Table 5. Incidence Rates of Common Cold According to the Age of Onset of Asthma at Preschool age, Primary School age, and Young Adulthood, Espoo Cohort Study, 1991–2011 [a]

Asthma Onset	No.	Age: 1–6 Years		Age: 7–13 Years		Age: 21–27 Years	
		IR (95% CI)	IRD (95% CI)	IR (95% CI)	IRD (95% CI)	IR (95% CI)	IRD (95% CI)
No asthma	2,253	2.57 (2.51, 2.64)	1.00	1.88 (1.82, 1.95)	1.00	2.11 (2.03, 2.18)	1.00
0 to 6 yrs. [b]	167	3.79 (3.50, 4.10)	1.22 (0.91, 1.52)	2.35 (2.10, 2.62)	0.47 (0.20, 0.73)	2.36 (2.08, 2.66)	0.25 (-0.04, 0.55)
7 to 13 yrs. [b]	93			2.41 (2.09, 2.77)	0.53 (0.19, 0.87)	2.58 (2.22, 2.97)	0.47 (0.09, 0.85)
14 to 27 yrs.	55					2.36 (1.97, 2.81)	0.26 (-0.16, 0.67)
Asthma ever	315	3.31 (3.11, 3.52)	0.74 (0.52, 0.95)	2. 29 (2.12, 2.48)	0.42 (0.22, 0.61)	2.43 (2.33, 2.63)	0.32 (0.11, 0.53)

IR – Incidence Rate per person-year; CI – Confidence interval; IRD – Incidence Rate difference per person-year.

[a] Incidence rate differences (IRDs) are calculated from IRs of subjects with the onset of asthma during different age periods subtracted by IRs among subjects without asthma during the corresponding age period.

[b] Onset of asthma before the occurrence of infections was applied in the analyses.

Web Table 6. Incidence Rates of Tonsillitis According to the Age of Onset of Asthma at Preschool age, Primary School age, and Young Adulthood, the Espoo Cohort Study 1991–2011 [a]

Asthma Onset	No.	Age: 1–6 Years		Age: 7–13 Years		Age: 21–27 Years	
		IR (95% CI)	IRD (95% CI)	IR (95% CI)	IRD (95% CI)	IR (95% CI)	IRD (95% CI)
No asthma	2,253	0.09 (0.08, 0.11)	1.00	0.13 (0.12, 0.15)	1.00	0.09 (0.08, 0.11)	1.00
0 to 6 yrs. [b]	167	0.1 (0.06, 0.16)	0.004 (-0.04, 0.05)	0.15 (0.09, 0.23)	0.01 (-0.05, 0.08)	0.14 (0.08, 0.22)	0.04 (-0.03, 0.11)
7 to 13 yrs. [b]	93			0.14 (0.07, 0.25)	0.01 (-0.07, 0.10)	0.03 (0.003, 0.1)	-0.07 (-0.11, -0.03)
14 to 27 yrs.	55					0.11 (0.04, 0.24)	0.01 (-0.07, 0.1)
Asthma ever	315	0.11 (0.08, 0.15)	0.01 (-0.02, 0.05)	0.14 (0.10, 0.19)	0.006 (-0.04, 0.05)	0.09 (0.06, 0.14)	-0.002 (-0.04, 0.04)

n – number of subjects; IR – Incidence Rate per person-year; CI – Confidence interval; IRD – Incidence Rate difference per person-year.
[a] Incidence rate differences (IRDs) are calculated from IRs of subjects with the onset of asthma during different age periods subtracted by IRs among subjects without asthma during the corresponding age period.
[b] Onset of asthma before the occurrence of infections was applied in the analyses.

Web Table 7. Incidence Rates of Otitis Media According to the Age of Onset of Asthma at Preschool age, Primary School age, and Young Adulthood, Espoo Cohort Study, 1991–2011[a]

Asthma Onset	No.	Age: 1–6 Years		Age: 7–13 Years		Age: 21–27 Years	
		IR (95% CI)	IRD (95% CI)	IR (95% CI)	IRD (95% CI)	IR (95% CI)	IRD (95% CI)
No asthma	2,253	0.95 (0.91, 0.99)	1.0	0.21 (0.19, 0.23)	1.0	0.05 (0.03, 0.06)	1.0
0 to 6 yrs. [b]	167	1.25 (1.09, 1.43)	0.29 (0.12, 0.47)	0.29 (0.21, 0.40)	0.08 (-0.01, 0.18)	0.13 (0.07, 0.21)	0.08 (0.01, 0.15)
7 to 13 yrs. [b]	93			0.29 (0.19, 0.43)	0.08 (-0.04, 0.20)	0.08 (0.03, 0.18)	0.04 (-0.03, 0.10)
14 to 27 yrs.	55					0.22 (0.11, 0.38)	0.17 (0.05, 0.30)
Asthma ever	315	1.07 (0.96, 1.19)	0.12 (-0.003, 0.24)	0.27 (0.21, 0.34)	0.06 (-0.004, 0.13)	0.13 (0.09, 0.19)	0.09 (0.04, 0.14)

n – number of subjects; IR – Incidence Rate per person-year; CI – Confidence interval; IRD – Incidence Rate difference per person-year.

[a] Incidence rate differences (IRDs) are calculated from IRs of subjects with the onset of asthma during different age periods subtracted by IRs among subjects without asthma during the corresponding age period.

[b] Onset of asthma before the occurrence of infections was applied in the analyses.

Web Table 8. Incidence Rates of Sinusitis According to the Age of Onset of Asthma at Preschool age, Primary School age, and Young Adulthood, Espoo Cohort Study, 1991–2011 [a]

Asthma Onset	No.	Age: 1–6 Years		Age: 7–13 Years		Age: 21–27 Years	
		IR (95% CI)	IRD (95% CI)	IR (95% CI)	IRD (95% CI)	IR (95% CI)	IRD (95% CI)
No asthma	2,253	0.05 (0.04, 0.06)	1.00	0.13 (0.11, 0.15)	1.00	0.20 (0.18, 0.23)	1.00
0 to 6 yrs. [b]	167	0.29 (0.22, 0. 39)	0.25 (0.16, 0.33)	0.45 (0.34, 0.57)	0.32 (0.20, 0.43)	0.40 (0.29, 0.53)	0.20 (0.08, 0.32)
7 to 13 yrs. [b]	93			0.46 (0.32, 0.63)	0.33 (0.18, 0.47)	0.23 (0.14, 0.37)	0.03 (-0.08, 0.15)
14 to 27 yrs.	55					0.64 (0.44, 0.89)	0.44 (0.22, 0.65)
Asthma ever	315	0.22 (0.17, 0.28)	0.17 (0.12, 0.23)	0.40 (0.33, 0.48)	0.27 (0.19, 0.35)	0.40 (0.33, 0.49)	0.20 (0.12, 0.28)

n – number of subjects; IR – Incidence Rate per person-year; CI – Confidence interval; IRD – Incidence Rate difference per person-year.

[a] Incidence rate differences (IRDs) are calculated from IRs of subjects with the onset of asthma during different age periods subtracted by IRs among subjects without asthma during the corresponding age period.

[b] Onset of asthma before the occurrence of infections was applied in the analyses.

Web Table 9. Incidence Rates of Bronchitis According to the Age of Onset of Asthma at Preschool age, Primary School age, and Young Adulthood, Espoo Cohort Study, 1991–2011[a]

Asthma Onset	No.	Age: 1–6 Years		Age: 7–13 Years		Age: 21–27 Years	
		IR (95% CI)	IRD (95% CI)	IR (95% CI)	IRD (95% CI)	IR (95% CI)	IRD (95% CI)
No asthma	2,253	0.20 (0.18, 0.21)	1.00	0.08 (0.07, 0.92)	1.00	0.09 (0.07, 0.11)	1.00
0 to 6 yrs. [b]	167	0.79 (0.67, 0.94)	0.60 (0.46, 0.74)	0.28 (0. 19, 0.38)	0.20 (0.11, 0.29)	0.19 (0.12, 0.29)	0.10 (0.02, 0.19)
7 to 13 yrs. [b]	93			0.22 (0.13, 0.35)	0.14 (0.04, 0.24)	0.29 (0.18, 0.45)	0.20 (0.07, 0.33)
14 to 27 yrs.	55					0.28 (0.16, 0.46)	0.18 (0.05, 0.33)
Asthma ever	315	0.57 (0.49, 0.66)	0.38 (0.29, 0.46)	0.22 (0.17, 0.29)	0.14 (0.09, 0.20)	0.24 (0.18, 0.31)	0.15 (0.09, 0.22)

n – number of subjects; IR – Incidence Rate per person-year; CI – Confidence interval; IRD – Incidence Rate difference per person-year.

[a] Incidence rate differences (IRDs) are calculated from IRs of subjects with the onset of asthma during different age periods subtracted by IRs among subjects without asthma during the corresponding age period.

[b] Onset of asthma before the occurrence of infections was applied in the analyses.

Web Table 10. Incidence Rates of Pneumonia According to the Age of Onset of Asthma at Preschool age, Primary School age, and Young Adulthood, Espoo Cohort Study, 1991–2011[a]

Asthma Onset	No.	Age: 1–6 Years		Age: 7–13 Years		Age: 21–27 Years	
		IR (95% CI)	IRD (95% CI)	IR (95% CI)	IRD (95% CI)	IR (95% CI)	IRD (95% CI)
No asthma	2,253	0.03 (0.02, 0.04)	1.00	0.02 (0.01, 0.03)	1.00	0.01 (0.006, 0.02)	1.00
0 to 6 yrs. [b]	167	0.16 (0.11, 0.24)	0.13 (0.07, 0.19)	0.03 (0.01, 0.07)	0.008 (-0.02, 0.04)	0.05 (0.02, 0.12)	0.04 (0.00, 0.09)
7 to 13 yrs. [b]	93			0.05 (0.01, 0.12)	0.03 (-0.02, 0.07)	0	-0.01 (-0.02, -0.005)
14 to 27 yrs.	55					0.13 (0.05, 0.26)	0.12 (0.02, 0.21)
Asthma ever	315	0.10 (0.07, 0.15)	0.07 (0.04, 0.11)	0.03 (0.02, 0.06)	0.01 (-0.01, 0.04)	0.05 (0.03, 0.09)	0.04 (0.01, 0.07)

n – number of subjects; IR – Incidence Rate per person-year; CI – Confidence interval; IRD – Incidence Rate difference per person-year.

[a] Incidence rate differences (IRDs) are calculated from IRs of subjects with the onset of asthma during different age periods subtracted by IRs among subjects without asthma during the corresponding age period.

[b] Onset of asthma before the occurrence of infections was applied in the analyses.

Web Table 11. Incidence Rates of Respiratory Tract Infections at Preschool age, Primary School age, and at Young Adulthood According to the Age of Onset of Asthma, Espoo Cohort Study, 1991–2011.

Asthma Onset	No.	Age: 1–6 Years		Age: 7–13 Years		Age: 21–27 Years	
		IR (95% CI)	IRD (95% CI)	IR (95% CI)	IRD (95% CI)	IR (95% CI)	IRD (95% CI)
**Upper respiratory tract infections**							
No asthma	2,253	3.67 (3.60, 3.75)	Ref.	2.35 (2.28, 3.43)	Ref.	2.45 (2.36, 2.53)	Ref.
0 to 6 yrs. [a]	167	5.44 (5.09, 5.80)	1.76 (1.40, 2.13)	3.23 (2.94, 3.55)	0.88 (0.57, 1.19)	3.02 (2.70, 3.36)	0.57 (0.24, 0.91)
7 to 13 yrs. [a]	93			3.30 (2.92, 3.72)	0.95 (0.55, 1.35)	2.92 (2.54, 3.34)	0.47 (0.07, 0.87
14 to 27 yrs.	55					3.33 (2.86, 3.85)	0.88 (0.39, 1.37)
Asthma ever	315	4.72 (4.48, 4.96)	1.04 (0.79, 1.29)	3.11 (2.90, 3.32)	0.75 (0.53, 0.98)	3.06 (2.84, 3.29)	0.61 (0.38, 0.85)
**Lower respiratory tract infections [b]**							
No asthma	2,253	22.64 (20.71, 24.69)	Ref.	9.84 (8.41, 11.44)	Ref.	9.54 (7.98, 11.31)	Ref.
0 to 6 yrs. [a]	167	95.81 (81.54, 111.86)	73.17 (58.20, 88.14)	30.66 (22.09, 41.44)	20.82 (11.43, 30.21)	23.42 (15.30, 34.32)	13.89 (4.74, 23.04)
7 to 13 yrs. [a]	93			26.51 (16.61, 40.13)	16.67 (5.49, 27.84)	27.39 (16.73, 42.31)	17.86 (5.74, 29.98)
14 to 27 yrs.	55					40.00 (25.07, 60.56)	30.46 (13.67, 47.26)
Asthma ever	315	67.94 (59.14, 77.67)	45.3 (35.99, 54.61)	25.56 (19.85, 32.41)	15.72 (9.47, 21.98)	28.45 (22.09, 36.07)	18.91 (11.96, 25.87)

n – number of subjects; IR – Incidence Rate per person-year; CI – Confidence interval; IRD – Incidence Rate Difference per person-year.

[a] Onset of asthma before the occurrence of infections was applied in the analyses.

[b] IR and IRD are presented per 100 person-years.

Web Table 12. Incidence Rates of Respiratory Tract Infections Requiring Hospitalization or Health Care Visit by the Age of Onset of Asthma, Espoo Cohort Study, 1991–2011(*n* = 2,568).

Asthma Onset	0–6 Years		7–13 Years		14–20 years		21–27 Years	
	IR (95% CI)	IRD (95% CI)	IR (95% CI)	IRD (95% CI)	IR (95% CI)	IRD (95% CI)	IR (95% CI)	IRD (95% CI)
	Upper Respiratory Tract Infections							
No asthma	1.12 (0.95, 1.31)	Ref.	0.29 (0.21, 0.4)	Ref.	0.92 (0.76, 1.09)	Ref.	0.26 (0.18, 0.36)	Ref.
0 to 6 yrs. [a]	5.59 (4.22, 7.26)	4.47 (3.00, 5.95)	0.79 (0.34, 1.57)	0.50 (-0.06, 1.06)	1.79 (1.06, 2.84)	0.88 (0.03, 1.72)	1.69 (0.99,2.72)	1.44 (0.63, 2.25)
7 to 13 yrs. [a]			0	-0.29 (-0.39, -0.20)	0.72 (0.19, 1.84)	-0.2 (-0.92, 0.52)	0.72 (0.19, 1.84)	0.46 (-0.25, 1.17)
14 to 27 yrs.					3.03 (1.45, 5.57)	2.11 (0.23, 3.99)	0.60 (0.07, 2.19)	0.35 (-0.49, 1.19)
Asthma ever	3.59 (2.79, 4.56)	2.48 (1.61, 3.35)	0.42 (0.18, 0.83)	0.13 (-0.18, 0.43)	1.69 (1.16, 2.39)	0.78 (0.17, 1.38)	1.22 (0.77, 1.83)	0.96 (0.45, 1.46)
	Lower Respiratory Tract Infections							
No asthma	0.42 (0.32, 0.55)	Ref.	0.18 (0.12, 0.27)	Ref.	0.54 (0.42, 0.68)	Ref.	0.15 (0.09, 0.23)	Ref.
0 to 6 yrs. [a]	4.69 (3.45, 6.24)	4.27 (2.92, 5.61)	0.39 (0.11, 1.02)	0.21 (-0.18, 0.61)	0.09 (0, 0.56)	-0.44 (-0.67, -0.21)	0.19 (0.02, 0.72)	0.05 (-0.23, 0.34)
7 to 13 yrs. [a]			0		0		0	
14 to 27 yrs.					0		0	
Asthma ever	2.91 (2.19, 3.79)	2.49 (1.71, 3.27)	0.21 (0.06, 0.54)	0.02 (-0.19, 0.25)	0.05 (0.001, 0.29)	-0.49 (-0.65, -0.33)	0.11 (0.01, 0.38)	-0.04 (-0.2, 0.12)

IR – Incidence rate per 100 person-year; CI – Confidence interval; IRD – Incidence rate difference per 100 person-year.

[a] Onset of asthma before the occurrence of infections was applied in the analyses.

Web Table 13. The Effect of Asthma on the Risk of Hospitalization or Health Care Visits due to Upper Respiratory Tract Infections from Childhood to Young Adulthood, Espoo Cohort Study, 1991–2011

Onset of Asthma	Timing of Infection (Age in Years)	Crude IRR (95% CI)	Adjusted IRR (95% CI) [a]
Never had asthma (*n* = 2,253)		1.00	1.00
Preschool age (0 – 6 yrs.) (*n* = 167) [b]	7 – 13	2.70 (0.76, 9.63)	2.91 (0.61, 13.86)
	14 – 20	1.96 (0.81, 4.75)	1.75 (0.69, 4.43)
	21 – 27	6.55 (1.41, 28.41)	6.10 (1.36, 27.44)
	7 – 27	2.70 (1.41, 5.18)	2.55 (1.26, 5.17)
Primary school age (7 – 13 yrs.) (*n* = 93) [b]	14 – 20	0.78 (0.20, 3.05)	0.732 (0.18, 2.93)
	21 – 27	2.77 (0.39, 19.61)	3.02 (0.45, 20.27)
	14 – 27	1.18 (0.39, 3.60)	1.20 (0.39, 3.66)
Asthma ever (0 – 27 yrs.) (*n* = 315)	0 – 6.	3.22 (2.06, 5.04)	2.96 (1.83, 4.78)
	7 – 13	1.43 (0.47, 4.36)	1.28 (0.34, 4.84)
	14 – 20	1.85 (0.95, 3.58)	1.78 (0.89, 3.58)
	21 – 27	4.70 (1.50, 14.71)	4.14 (1.24, 13.77)
	0 – 27	2.68 (1.91, 3.75)	2.50 (1.74, 3.59)

IRR – Incidence rate ratio; CI – Confidence interval

[a] The models were adjusted for sex, personal atopy, maternal smoking, family socioeconomic status, parental atopy/asthma, second-hand smoke exposure, indoor mold exposure at baseline, duration of pregnancy, and breastfeeding.

[b] Onset of asthma before the occurrence of infections was applied in the analyses.

Web Table 14. The Effect of Asthma on the Risk of Hospitalization or Health Care Visits due to Lower Respiratory Tract Infections from Childhood to Young Adulthood, Espoo Cohort Study, 1991–2011

**Onset of Asthma**	**Timing of Infection (Age in Years)**	**Crude IRR (95% CI)**	**Adjusted IRR (95% CI)** [a]
Never had asthma (*n* = 2,253)		1.00	1.00
Preschool age (0 – 6 yrs.) (*n* = 167) [b]	7 – 13 yrs.	2.16 (0.33, 13.97)	2.34 (0.32, 17.01)
	14 – 20 yrs.	0.18 (0.02, 2.11)	0.14 (0.01, 1.88)
	21 – 27 yrs.	1.35 (0.09, 21.18)	0.66 (0.04, 11.12)
	7 – 27 yrs.	0.94 (0.26, 3.54)	0.78 (0.20, 3.08)
Primary school age (7 – 13 yrs.) (*n* = 93) [b]	14 – 20 yrs.	NA	NA
	21 – 27 yrs.	NA	NA
	14 – 27 yrs.	NA	NA
Asthma ever (0 – 27 yrs.) (*n* = 315)	0 – 6 yrs.	6.90 (4.09, 11.64)	6.87 (3.99, 11.82)
	7 – 13 yrs.	1.14 (0.23, 5.71)	0.82 (0.12, 5.46)
	14 – 20 yrs.	0.10 (0.01, 0.93)	0.07 (0.01, 0.81)
	21 – 27 yrs.	0.72 (0.07, 7.26)	0.33 (0.02, 5.14)
	0 – 27 yrs.	4.43 (2.76, 7.13)	4.40 (2.66, 7.26)

IRR – Incidence rate ratio; CI – Confidence interval; NA – Not applicable, due to few or zero cases per category of the exposure variable.

[a] The models were adjusted for sex, personal atopy, maternal smoking, family socioeconomic status, parental atopy/asthma, second-hand smoke exposure, indoor mold exposure at baseline, duration of pregnancy, and breastfeeding

[b] Onset of asthma before the occurrence of infections was applied in the analyses.
